# Interplay between the phosphatase PHLPP1 and E3 ligase RNF41 stimulates proper kinetochore assembly via the outer-kinetochore protein SGT1

**DOI:** 10.1074/jbc.M117.782896

**Published:** 2017-07-10

**Authors:** Narmadha Reddy Gangula, Subbareddy Maddika

**Affiliations:** From the Laboratory of Cell Death and Cell Survival, Centre for DNA Fingerprinting and Diagnostics, Nampally, Hyderabad 500001, India

**Keywords:** cell cycle, E3 ubiquitin ligase, kinetochore, mitosis, phosphatase, PHLPP1, RNF41, SGT1

## Abstract

Kinetochores link chromosomes to spindle microtubules and are essential for accurate chromosome segregation during cell division. Kinetochores assemble at the centromeric region of chromosomes as a multiprotein complex. However, the molecular mechanisms of kinetochore assembly have not yet been fully elucidated. In this study, we identified pleckstrin homology domain and leucine-rich repeat protein phosphatase 1 (PHLPP1) as a regulatory phosphatase that facilitates proper kinetochore assembly. We found that PHLPP1 interacted with the essential outer-kinetochore protein SGT1 and stabilized its protein levels. Loss of PHLPP1 from cells led to SGT1 degradation and thereby caused defective kinetochore assembly. We also found that the ring finger protein 41 (RNF41) as an E3 ligase ubiquitinated and degraded SGT1 in a phosphorylation-dependent manner. PHLPP1 dephosphorylated SGT1 at four conserved residues (Ser-17, Ser-249, Ser-289, and Thr-233) and thereby prevented SGT1 from associating with RNF41, in turn, countering SGT1 degradation. Importantly, depletion of RNF41 or expression of a non-phosphorylatable SGT1 mutant rescued the kinetochore defects caused by the loss of PHLPP1. Taken together, our results suggest that PHLPP1 plays an important role in the assembly of kinetochores by counteracting RNF41-mediated SGT1 degradation.

## Introduction

During cell division, accurate chromosome segregation is dependent on correct attachment of chromatids to spindle apparatus (1). Kinetochores are the protein structures assembled on chromosomes where spindle fibers attach during cell division to pull sister chromatids apart for proper segregation of chromosomes (2, 3). The kinetochore also monitors inaccurate chromosome-spindle attachments, thus preventing anaphase entry until all chromosomes are correctly aligned, through spindle assembly checkpoint (4). Kinetochore composed of nearly 100 different proteins is distributed in three distinct protein layers, an inner core kinetochore, an outer kinetochore, and a fibrous corona (3, 5). The core kinetochore containing mainly the histone variants such as CEPN-A and auxiliary proteins is constitutively present at the centromeric DNA throughout the cell cycle. The outer kinetochore region that contains proteins such as CENP-E, CENP-F, HEC1, and others is dynamically assembled during specific stages of mitosis to mainly interact with the spindle microtubules. The outermost regions of the kinetochore form a fibrous corona, which mainly consists of the components that are involved in the spindle assembly checkpoint. Given the large number of kinetochore components, it is essential for the cell to have strong regulatory machinery to ensure their proper assembly and function. Although the functions of many of these individual components have been elucidated, the regulatory mechanisms for their assembly is still far from complete.

Here, we identified PHLPP1 (pleckstrin homology domain leucine-rich repeat protein phosphatase 1) as an essential protein required for proper assembly of kinetochores in cells. PHLPP1 is a highly conserved serine/threonine phosphatase, which belongs to PPM family of phosphatases (6). PHLPP1 functions as a tumor suppressor by acting on distinct cellular substrates such as Akt, PKC, S6K, RAF1, and Mst1 (7–10). PHLPP1 dephosphorylates Akt on the Ser-473 residue and thereby negatively regulates its function in cell survival and proliferation (11). PHLPP1 also dephosphorylates Mst1 on the Thr-387 site, which activates Mst1 and its downstream effectors to induce apoptosis (10). In contrast, PHLPP1-mediated dephosphorylation of S6 kinase 1 on Thr-389 is shown to inhibit protein translation and cell growth (9). Besides its role in cell proliferation and survival, studies on functions of PHLPP1 in other cellular processes are very limited.

In our earlier study (12), we found SGT1, an outer kinetochore protein, in the list of PHLPP1-associated proteins. SGT1 (suppressor of G2 allele of SKP1 homolog), a highly conserved protein from yeast to humans, acts as a co-chaperone of Hsp90. Functional studies using yeast and cultured human cells have established SGT1 as an essential outer kinetochore protein that is required for its assembly (13–15). SGT1-depleted cells have compromised kinetochore assembly and display severe mitotic defects such as centrosome defects, alterations in spindle, and chromosome alignment (15, 16). However, the mechanisms that regulate SGT1 functions are largely unknown.

In this study, we found that SGT1 protein levels are tightly controlled in cells by RNF41-mediated ubiquitination. RNF41 (also known as NRDP1) is a ring type E3 ubiquitin ligase that has been implicated in controlling various cellular pathways, such as the EGF receptor, cytokine receptor, and T cell receptor signaling through ubiquitination of different substrates in these pathways (17–19). However, its role in regulating kinetochore proteins has not been documented so far. We found that RNF41 degrades SGT1 in a phosphorylation-dependent manner. PHLPP1 dephosphorylates SGT1 and thus prevents RNF41-mediated SGT1 degradation. In summary, we demonstrated that PHLPP1 maintains optimal SGT1 protein levels during early mitosis by counteracting RNF41-mediated degradation of SGT1 and thereby facilitates proper kinetochore assembly.

## Results

### PHLPP1 interacts with SGT1 and is required for kinetochore assembly

**Figure 1.**
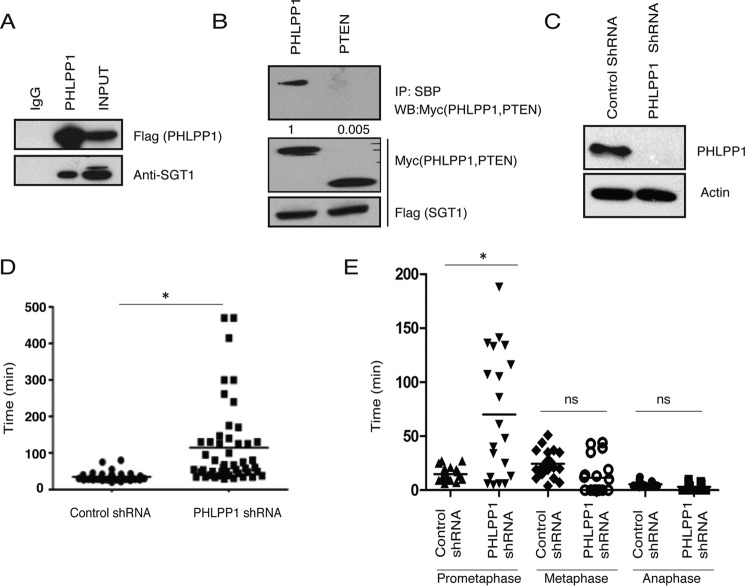
**PHLPP1 regulates mitotic progression by interacting with SGT1.** *A,* HEK293T cell lysate expressing triple-tagged SFB-PHLPP1 was subjected to immunoprecipitation with either IgG or FLAG antibody, and its interaction with endogenous SGT1 was detected by immunoblotting with SGT1 antibody. *B,* HEK293T cell lysate expressing SFB-SGT1 along with either Myc-PHLPP1 or Myc-PTEN was subjected to immunoprecipitation (*IP*) with SBP beads, and their interaction was detected by immunoblotting with Myc antibody. Normalized data from IP/total protein derived from the quantification of the blots by using Image Lab software are shown. WB, Western blotting. *C,* PHLPP1 was depleted in HeLa cells by using shRNA. *D,* transition of cells through mitosis was analyzed by live cell time-lapse microscopy after synchronizing cells using double thymidine block. Time taken by each cell from mitotic entry to separation of cells after cytokinesis was calculated, and the data were plotted for control and PHLPP1-depleted cells (*n* = 50), *p* < 0.05. *E,* U2OS cells stably expressing H2B-mCherry were analyzed by live cell time-lapse microscopy. Time spent by each cell in different stages of mitosis was calculated, and the data were plotted for control and PHLPP1-depleted cells (*n* = 20). *ns*, not significant. *, *p* < 0.05, Student's *t* test.

In an attempt to identify new cellular functions of PHLPP1, we analyzed the list of its interacting proteins derived from biochemical purification followed by proteomic analysis in our earlier study (12). We found SGT1, an outer kinetochore protein, as one of the potential interacting partners of PHLPP1. We hypothesized that PHLPP1 may function in kinetochore assembly by interacting with SGT1. By performing immunoprecipitation followed by immunoblotting with specific antibodies, we confirmed the association of endogenous SGT1 with PHLPP1 (Fig. 1*A*). Furthermore, we found that SGT1 interacts with PHLPP1 but not with phosphatase and tensin homolog, another phosphatase in the PI3K/AKT pathway thus validating the specificity of the PHLPP1–SGT1 interaction (Fig. 1*B*).

**Figure 2.**
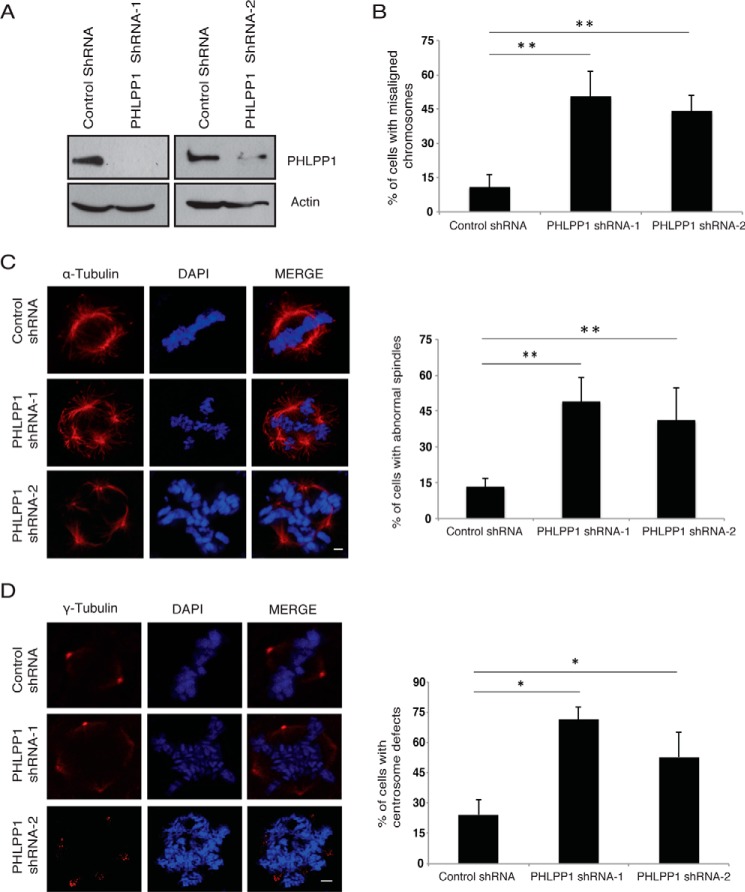
**Depletion of PHLPP1 leads to mitotic defects.** HeLa cells were transfected with control and two different PHLPP1 shRNAs (*A*) and then subjected to thymidine treatment and followed by immunofluorescence staining to assess the percentage of cells with misaligned chromosomes(*B*). *C,* HeLa cells were transfected with control and PHLPP1 shRNAs, and 24 h after transfection cells were treated with thymidine and then processed for immunofluorescence staining with α-tubulin antibody to check the spindle defects. *D,* γ-tubulin antibody was used for centrosome defects (*scale bar,* 2 μm). Quantification of data is shown on *right* (*n* = 50 cells each). **, *p* < 0.01; *, *p* < 0.05, Student's *t* test.

**Figure 3.**
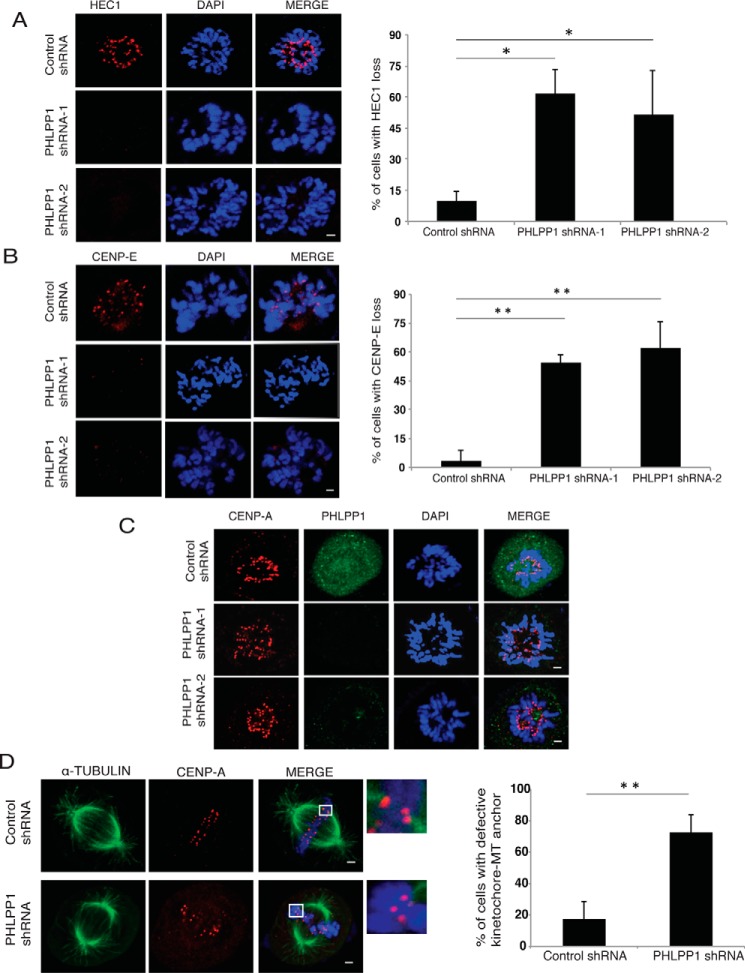
**PHLPP1 facilitates kinetochore assembly.** *A,* localization of outer kinetochore protein HEC1. *B,* CENP-E to kinetochores was tested in control and PHLPP1-depleted cells by using immunofluorescence (*scale bar,* 2 μm). Quantification of cells with defective localization is shown on *right* (*n* = 50 cells each). **, *p* < 0.01; *, *p* < 0.05, Student's *t* test. *C,* localization of inner kinetochore protein CENP-A was tested in control and PHLPP1-depleted cells by using immunofluorescence (*scale bar,* 2 μm). *D,* microtubules (*MT*) and kinetochores were stained using α-tubulin and CENP-A antibodies, respectively, and the attachment of MTs with kinetochores in control and PHLPP1-depleted cells was assessed (*scale bar,* 2 μm). Quantification of cells with defective MT-kinetochore anchoring is shown on *right* (*n* = 50 cells each). **, *p* < 0.01, Student's *t* test.

Because SGT1 is critical for proper kinetochore assembly during the mitotic cycle, we next tested whether loss of PHLPP1 phenocopies SGT1 loss from cells. Time-lapse imaging revealed that silencing of PHLPP1 in HeLa cells (Fig. 1*C*) leads to delayed progression of cells in mitosis (Fig. 1*D*). Although control cells complete mitosis within 60 min after rounding up, cells expressing PHLPP1 shRNA spent several hours in mitosis before they aberrantly exit without dividing or undergoing cell death. Furthermore, live cell analysis using U2OS cells stably expressing H2B mCherry revealed that PHLPP1-depleted cells are predominantly delayed in the prometaphase stage of mitosis (Fig. 1*E*). Delayed progression of cells in mitosis upon PHLPP1 depletion (Fig. 2*A*) is accompanied by multiple severe mitotic defects such as misaligned chromosomes (Fig. 2*B*), multipolar spindles (Fig. 2*C*), and abnormal centrosomes (Fig. 2*D*). Next, we tested the effect of PHLPP1 depletion on direct assembly of kinetochores by staining with specific markers. By using confocal immunofluorescence imaging, we found that outer kinetochore proteins such as HEC1 (Fig. 3*A*) and CENP-E (Fig. 3*B*) failed to localize to kinetochores in PHLPP1-depleted cells. In contrast, localization of core inner kinetochore protein CENP-A is unaffected by PHLPP1 loss (Fig. 3*C*). As depletion of PHLPP1 caused severe reduction in recruitment of outer kinetochore proteins, we next tested whether kinetochore–microtubule attachment is affected in these cells. Indeed, co-staining of kinetochores and microtubules with CENP-A and α-tubulin, respectively, revealed that PHLPP1 depletion led to defective attachment of microtubules with kinetochores (Fig. 3*D*). Thus, together these data suggest that PHLPP1 is necessary for proper assembly of kinetochores.

### PHLPP1 is required for maintaining SGT1 stability

**Figure 4.**
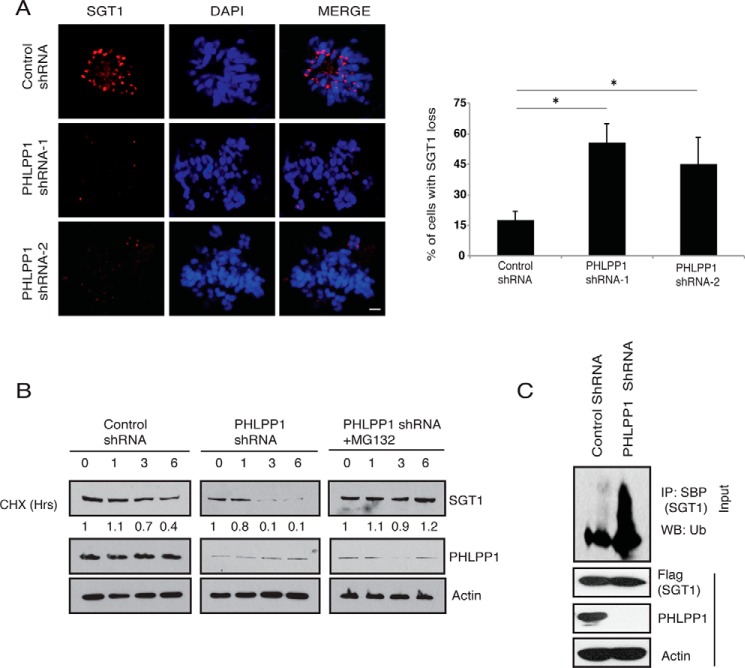
**PHLPP1 regulates SGT1 protein stability.** *A,* SGT1 levels at kinetochores in control and PHLPP1 shRNA-expressing cells were detected by immunofluorescence with SGT1-specific antibody (*scale bar,* 2 μm). Quantification of data is shown on *right* (*n* = 50 cells each). *, *p* < 0.05, Student's *t* test. *B,* HeLa cells were transfected with control and PHLPP1 shRNA. and 72 h post-transfection cells were treated with cycloheximide (*CHX*), and the protein levels of SGT1 were detected using SGT1 antibody at the indicated time points. Normalized data from SGT1/actin derived from the quantification of the blots by using Image Lab software is shown. *C,* cells transfected with control or PHLPP1 shRNA were treated with MG132 (10 μm) for 6 h, and the levels of SGT1 ubiquitination were detected using anti-ubiquitin (*Ub*) antibody after immunoprecipitating (*IP*) with streptavidin beads under denaturing conditions. *WB*, Western blotting.

To further understand how PHLPP1 participates in kinetochore assembly by interacting with SGT1, we tested SGT1 localization on kinetochores. Immunofluorescence studies suggested that upon PHLPP1 depletion SGT1 is lost from the kinetochores (Fig. 4*A*). This result prompted us to test whether SGT1 protein stability is controlled by PHLPP1. In fact, in a cycloheximide chase experiment, shRNA-mediated knockdown of PHLPP1 reduced the protein half-life of SGT1 to a greater extent suggesting that PHLPP1 is required for maintaining the stability of SGT1 (Fig. 4*B*). The reduction in SGT1 levels upon PHLPP1 depletion was rescued in the presence of the proteasome inhibitor MG132. Because polyubiquitination is known to regulate protein stability through the proteasome, we further tested whether PHLPP1 maintains SGT1 stability by controlling ubiquitination. Knockdown of PHLPP1 resulted in accumulation of polyubiquitinated SGT1 (Fig. 4*C*), suggesting that PHLPP1 is required for preventing SGT1 ubiquitination.

### RNF41 is an E3 ligase for SGT1

**Figure 5.**
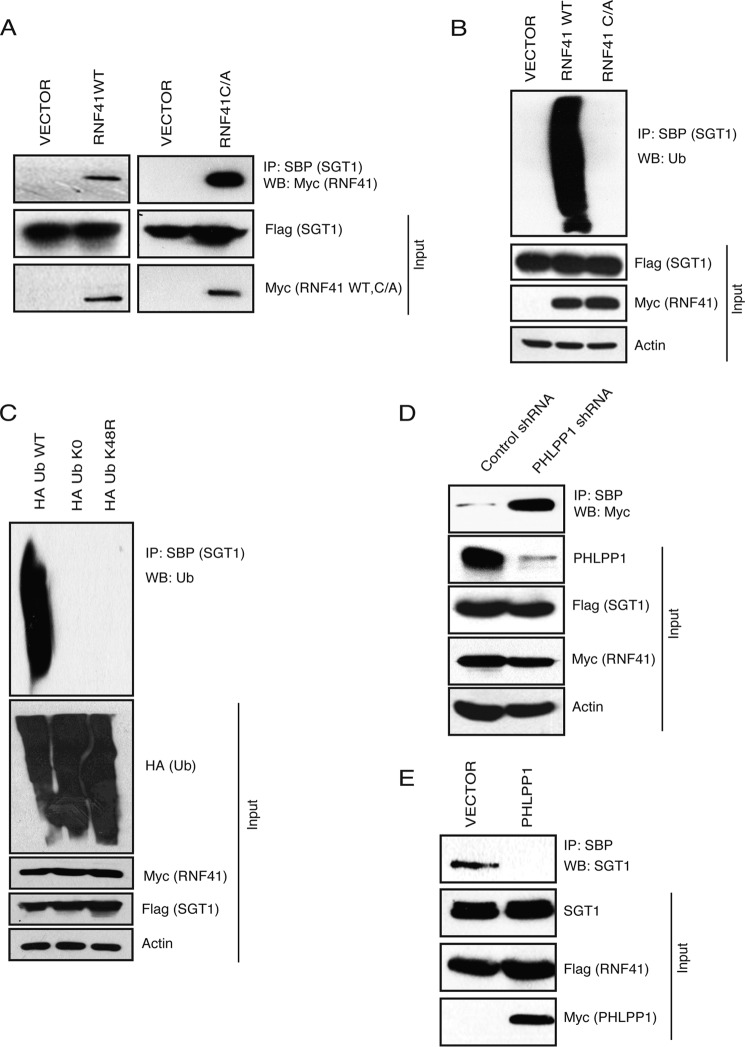
**PHLPP1 stabilizes SGT1 by preventing its association with RNF41.** *A,* HEK293T cells were transfected with SFB-tagged SGT1 along with Myc-tagged wild-type (*WT*) RNF41 or catalytically inactive mutant C34A (*C/A*), and their interaction was tested by immunoprecipitation (*IP*) with streptavidin beads followed by immunoblotting using Myc antibody. *WB,* Western blotting. *B,* 293T cells were transfected with SFB SGT1 along with either Myc RNF41 wild type (*WT*) or catalytically inactive mutant (*C/A*), and SGT1 ubiquitination was evaluated using anti-ubiquitin (*Ub*) antibody. *C,* 293T cells were transfected with HA Ub wild type, Ub K0, and Ub K48R and the ubiquitination of SGT1 was detected by immunoblotting with anti-ubiquitin antibody. *D,* cells were transduced with either control shRNA or PHLPP1 shRNA, and the interaction of RNF41 and SGT1 in these cells was tested by immunoprecipitation as indicated. *E,* cells were transfected with vector control or Myc-tagged PHLPP1, and the interaction of triple-tagged SFB-RNF41 with endogenous SGT1 in these cells was detected by immunoprecipitation with streptavidin beads followed by immunoblotting with SGT1 antibody.

Although we found that PHLPP1 controls SGT1 polyubiquitination, the enzymatic machinery that mediates SGT1 ubiquitination in the cells is unknown. Thus, next we sought to identify E3 ubiquitin ligase for SGT1. In an attempt to identify the E3 ligases for SGT1, we established a 293T derivative cell line expressing a triple-epitope (S-protein, FLAG, and streptavidin-binding peptide (SBP)^3^)-tagged version of SGT1 (SFB-SGT1). Tandem affinity purification of SGT1 using streptavidin-agarose beads and S-protein-agarose beads followed by mass spectrometry analysis revealed several SGT1-associated proteins (supplemental Table S1). Among these, we found RNF41 as a potential E3 ligase interacting with SGT1. We validated the interaction of SGT1 with wild-type (WT) RNF41 as well as a catalytically inactive mutant of RNF41 (C34A) in cells through co-immunoprecipitation experiments (Fig. 5*A*). Next, to test whether RNF41 can ubiquitinate SGT1, we performed ubiquitination assay in cells by co-expressing SFB SGT1 along with Myc-tagged RNF41 wild type and also the catalytically inactive mutant of RNF41. We observed that SGT1 was polyubiquitinated by wild-type RNF41 but not the catalytically inactive mutant (Fig. 5*B*). Polyubiquitination of SGT1 is through degradative Lys-48-linked ubiquitin chains as RNF41 failed to ubiquitinate SGT1 in the presence of ubiquitin K48R mutant (Fig. 5*C*). Because we identified RNF41 as an E3 ligase for SGT1 and PHLPP1 impedes SGT1 polyubiquitination, we hypothesized that PHLPP1 might prevent SGT1 from interacting with its E3 ligase RNF41. To test this hypothesis and to understand the interplay between RNF41 and PHLPP1 in regulating SGT1, co-immunoprecipitation was performed either after depleting PHLPP1 from cells or by exogenous expression of PHLPP1. Interaction of SGT1 with RNF41 is dramatically enhanced in the absence of PHLPP1 (Fig. 5*D*), and conversely exogenous expression of PHLPP1 leads to loss of SGT1 interaction with its E3 ligase (Fig. 5*E*). Thus, PHLPP1 protects SGT1 from polyubiquitination and degradation by interfering with SGT1 interaction with its E3 ligase RNF41.

### PHLPP1 dephosphorylates SGT1 and prevents its association with RNF41

**Figure 6.**
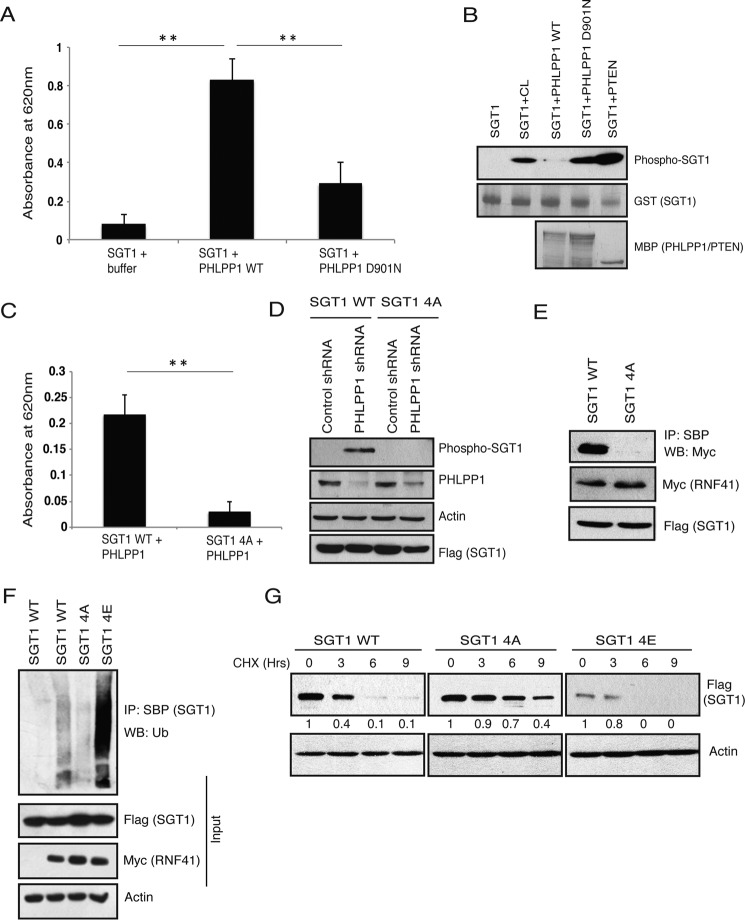
**Dephosphorylation of SGT1 by PHLPP1 is required for its stability.** *A, in vitro* phosphorylated SGT1 was incubated with purified wild type (*WT*) or inactive D901N mutant of PHLPP1 from HEK293T cells, and the released phosphate was detected by absorbance at 620 nm after adding Malachite Green reagent, (*n* = 3 independent experiments), *p* < 0.01. *B,* bacterially purified GST SGT1 was subjected to *in vitro* kinase assay using HEK293T cell lysate (*CL*), and then the phosphorylated proteins were incubated with either bacterially purified MBP-tagged wild-type (*WT*) PHLPP1, D901N mutant, or PTEN. The phosphorylation status of recombinant SGT1 was detected by immunoblotting with pIMAGO reagent after purifying SGT1 with glutathione-Sepharose beads. Non-phosphorylated SGT1 was used in the *1st lane* to show the specificity of pIMAGO reagent in detecting the phosphorylated proteins. *C,* bacterially purified GST SGT1 and SGT1 4A (S17A/T233A/S249A/S289A) mutants were subjected to *in vitro* kinase assay using HEK293T cell lysate, and then the phosphorylated proteins were incubated with bacterially purified MBP-tagged PHLPP1. The released phosphate was measured by absorbance at 620 nm after adding Malachite Green reagent (*n* = 3 independent experiments), *p* < 0.01. *D,* HeLa cells expressing either control or PHLPP1 shRNA were transfected with SFB-tagged SGT1 wild type (*WT*) and SGT1 4A mutant. The phosphorylation status of SGT1 in these cells was detected by immunoblotting with pIMAGO reagent after immunoprecipitating SGT1 with streptavidin beads. *E,* HEK293T cells were transfected with SFB SGT1 WT and SGT1 4A along with Myc RNF41, and their interaction was tested by immunoblotting with Myc antibody after immunoprecipitation (*IP*) with streptavidin beads. *WB*, Western blotting. *F,* 293T cells co-transfected with SFB SGT1 WT, SGT1 4A, SGT1 4E (S17E/T233E/S249E/S289E), and Myc RNF41 were treated with MG132, and the levels of SGT1 ubiquitination were detected. *G,* 293T cells expressing SGT1 WT, SGT1 4A, and SGT1 4E were treated with cycloheximide (*CHX*) after 24 h of transfection, and their protein levels were detected at the indicated times after immunoblotting with anti-FLAG antibody. Normalized data from SGT1/actin derived from the quantification of the blots by using Image Lab software is shown.

To understand the mechanistic details of how PHLPP1 prevents SGT1 from interaction with RNF41, we next tested whether SGT1 acts as a substrate of PHLPP1. By using an *in vitro* phosphatase assay, we found that wild-type PHLPP1, but not the PHLPP1 phosphatase-inactive mutant (D901N), readily dephosphorylated SGT1 (Fig. 6*A*). Furthermore, by using an *in vitro* pIMAGO-based detection of phosphorylation on recombinant proteins, we found that active PHLPP1, but not PTEN, dephosphorylates SGT1 thus confirming the specificity of PHLPP1-mediated dephosphorylation (Fig. 6*B*). By using mass spectrometric analysis, we found that human SGT1 was phosphorylated at five different residues (Ser-17, Ser-249, Ser-286, Ser-289, and Thr-233) in the cell (supplemental Fig. S1). We mutated each of these residues to alanine and tested the ability of PHLPP1 to dephosphorylate SGT1. We did not find any significant differences in the phosphate release by PHLPP1 using SGT1 single residue mutants. We assumed that PHLPP1 dephosphorylates multiple residues on SGT1. To test this possibility, we made several SGT1 mutants with mutations at these five residues in different combination. Indeed, we found that mutation of four residues Ser-17, Ser-249, Ser-289, Thr-233 (SGT1 4A) significantly lowered the release of phosphate from SGT1 by PHLPP1 (Fig. 6*C*). Furthermore, we confirmed the dephosphorylation of these sites in cells by expressing a SGT1 4A mutant in the presence of PHLPP1 shRNA. Although depletion of PHLPP1 caused enhanced phosphorylation of wild-type SGT1, no changes in the phosphorylation status of 4A mutant were observed (Fig. 6*D*).

Next, we tested whether phosphorylation of these residues is required for SGT1-RNF41 interaction, which is opposed by PHLPP1. Our co-immunoprecipitation experiments suggested that wild type, but not a phospho-dead 4A mutant, of SGT1 interacts with RNF41 (Fig. 6*E*). Furthermore, RNF41 could not ubiquitinate a phospho-dead SGT1 4A mutant, but a significant accumulation of polyubiquitinated protein was observed with phosphomimetic SGT1 4E mutant (all four residues mutated to glutamic acid) (Fig. 6*F*). Consistent with this, in a cycloheximide chase experiment, we observed that SGT1 4A mutant levels were stabilized compared with wild type, but on the other hand, the SGT1 4E mutant was highly unstable (Fig. 6*G*). Together, these results fully support our hypothesis that RNF41 interacts with SGT1 in a phosphorylated state, and PHLPP1 opposes RNF41 activity toward SGT1 by dephosphorylation.

### RNF41 depletion rescues the kinetochore defects caused by PHLPP1 loss

**Figure 7.**
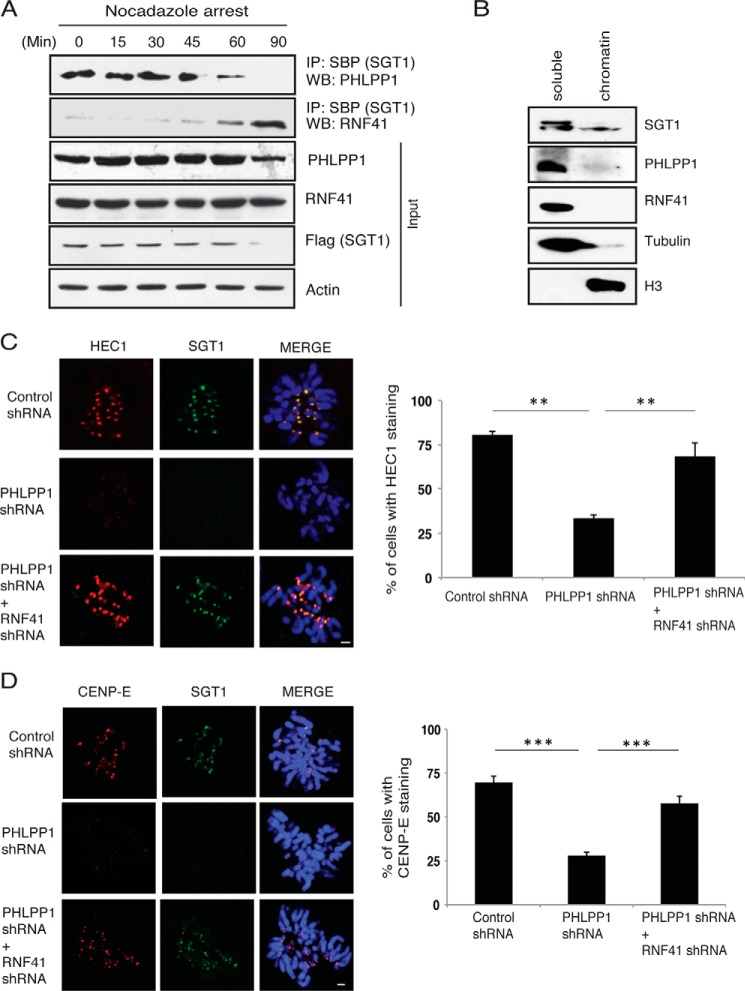
**Functional interplay between PHLPP1 and RNF41 during kinetochore formation.** *A,* HeLa cells expressing SFB-tagged SGT1 were treated with nocodazole, and later cells were collected at the indicated different times post-release. SGT1 interaction with PHLPP1 and RNF41 during different mitotic stages was tested by immunoblotting with respective antibodies after immunoprecipitation (*IP*) with streptavidin beads. *WB*, Western blotting. *B,* soluble and chromatin fractions were isolated by acid extraction, and localization of SGT1, PHLPP1, and RNF41 was detected by using specific antibodies. Histone H3 and tubulin were used as specific fraction markers. *C,* localization of HEC1 to kinetochores was tested in cells either expressing control shRNA, PHLPP1 shRNA, or co-expressing PHLPP1 and RNF41 shRNA (*scale bar,* 2 μm). Quantification of cells with defective HEC1 localization is shown on *right* (*n* = 50 cells each). **, *p* < 0.01, Student's *t* test. *D,* localization of CENP-E to kinetochores in these cells was tested by immunofluorescence (*scale bar,* 2 μm). Quantification of cells with defective CENP-E localization is shown on *right* (*n* = 50 cells each). ***, *p* < 0.001, Students's *t* test.

**Figure 8.**
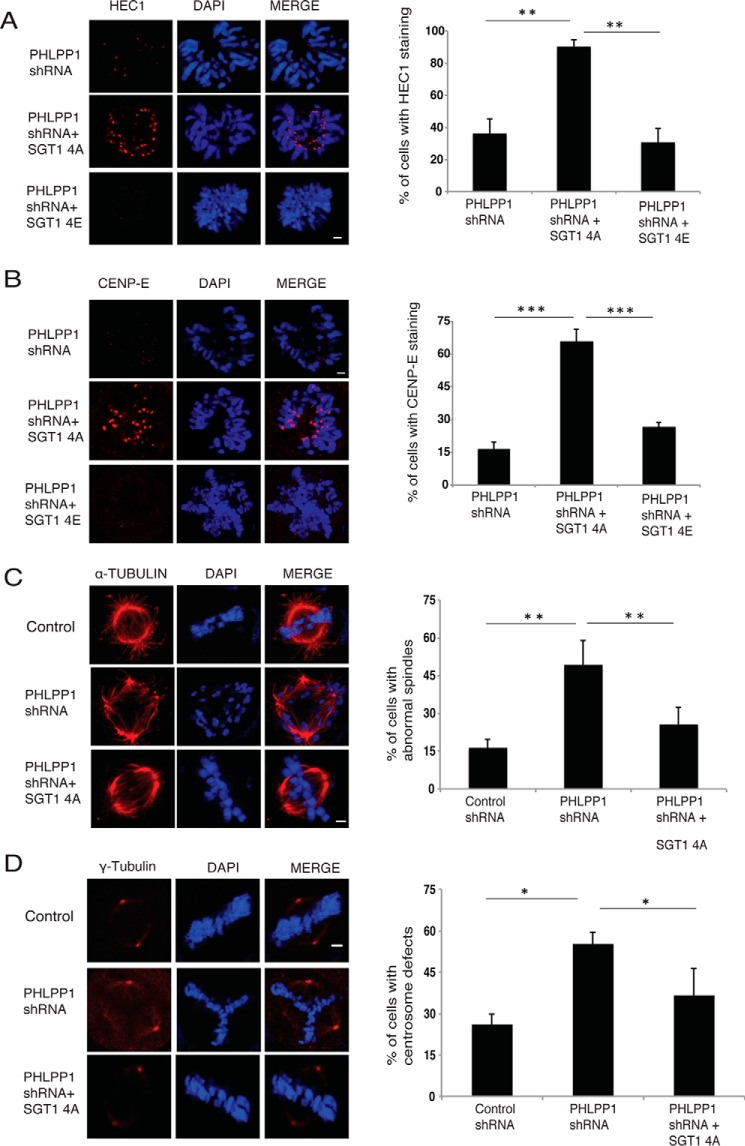
**Non-phosphorylatable mutant of SGT1 rescues mitotic defects.** *A,* cells expressing PHLPP1 shRNA were transfected with a phospho-dead SGT1 4A mutant or phospho-mimetic SGT1 4E mutant. Localization of HEC1 to kinetochores in these cells was tested by immunofluorescence (*scale bar,* 2 μm). Quantification of cells with defective HEC1 localization is shown on *right* (*n* = 50 cells each). **, *p* < 0.01, Student's *t* test. *B,* localization of CENP-E to kinetochores was also tested in these cells by immunofluorescence (*scale bar,* 2 μm). Quantification of cells with defective CENP-E localization is shown on right (*n* = 50 cells each). ***, *p* < 0.001, Student's *t* test. *C,* cells expressing PHLPP1 shRNA were transfected with a phospho-dead SGT1 4A mutant and then processed for immunofluorescence staining with α-tubulin antibody to check the spindle defects. *D,* γ-tubulin antibody for centrosome defects (*scale bar,* 2 μm). Quantification of the data is shown on *right* (*n* = 50 cells each). *, *p* < 0.05; **, *p* < 0.01, Student's *t* test.

To understand whether there exists a fine interplay between PHLPP1 and RNF41 in regulating SGT1 stability during mitotic stages, we arrested cells in mitosis using nocodazole and tested for SGT1–PHLPP1–RNF41 interaction. In the early stages of mitosis where kinetochore assembly occurs, PHLPP1 efficiently interacted with SGT1 and prevented its interaction with RNF41 thereby stabilizing SGT1 levels (Fig. 7*A*). During the later stages of mitosis, post-kinetochore assembly, interaction between PHLPP1 and SGT1 is lost, which then leads to RNF41 association and degradation of SGT1. The regulation of SGT1 by PHLPP1 and RNF41 appears to occur in the soluble pool of the cell but not on chromatin, because we did not observe any localization of RNF41 and PHLPP1 to chromatin (Fig. 7*B*). To further test whether the existence of interplay between PHLPP1 and RNF41 is functionally relevant for kinetochore assembly, we performed double depletion experiments. We hypothesized that SGT1 loss and kinetochore defects caused by PHLPP1 depletion would be rescued by simultaneous depletion of RNF41. In support of this hypothesis, co-depletion of RNF41 and PHLPP1 rescued SGT1 loss as well as recruitment of other proteins such as HEC1 (Fig. 7*C*) and CEPN-E (Fig. 7*D*) at kinetochores. In addition, overexpression of the non-phosphorylatable SGT1 4A mutant, but not phosphomimetic SGT1 4E mutant, also rescued the outer kinetochore assembly defects (Fig. 8, *A* and *B*), thus fully supporting our data that PHLPP1-mediated dephosphorylation of SGT1 is critical for proper kinetochore assembly. Interestingly, expression of the SGT1 4A mutant also rescued spindle defects (Fig. 8*C*) as well as centrosome defects (Fig. 8*D*) caused by PHLPP1 depletion, possibly suggesting that SGT1 is a major player in PHLPP1 controlled mitotic processes. Indeed, these data are in agreement with earlier studies where SGT1 was shown to be required for centrosome organization (16), and depletion of SGT1 leads to multiple mitotic defects such as spindle and centrosome defects along with kinetochore assembly defects (15).

**Figure 9.**
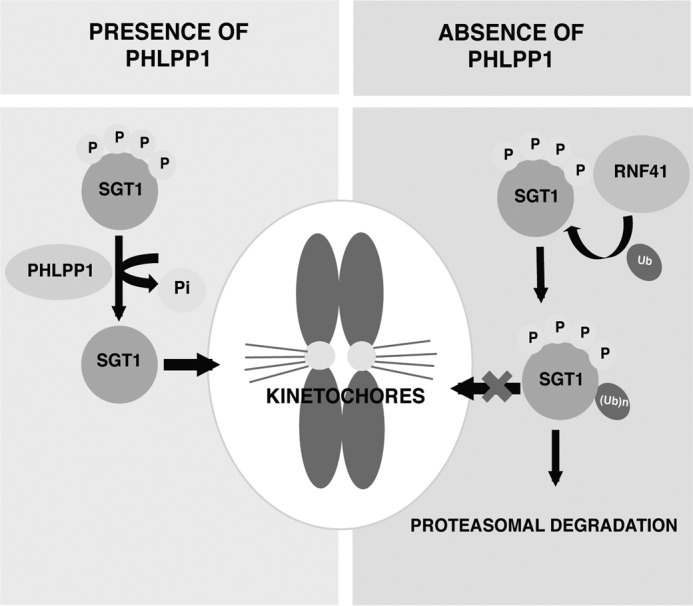
**Model showing the interplay between PHLPP1 and RNF41 in regulation of SGT1 at kinetochores.** PHLPP1 dephosphorylates SGT1 and maintains stable SGT1 at kinetochores. In the absence of PHLPP1, E3 ligase RNF41 associates with phosphorylated SGT1 and promotes its degradation and thereby limits SGT1 availability at kinetochores.

In conclusion, we demonstrated the existence of a dynamic interplay between PHLPP1 and RNF41 in regulation SGT1 stability, which is critical for proper kinetochore assembly (Fig. 9).

## Discussion

Kinetochore assembly and disassembly are highly dynamic processes that occur during cell division. Although inner kinetochore proteins such as CCAN (constitutive-centromere-associated network) persist at centromeres during the entire cell cycle, the outer kinetochore proteins such as the KMN network that is composed of KNL1, the Mis12 complex, and the NDC80 complex are recruited specifically during G_2_ and early mitotic phases of cell division. Proper recruitment of the outer kinetochore components is essential for correct chromosome segregation. Given the challenging task of reorganizing the outer kinetochore with nearly 40 proteins during a very short period of G_2_/M transition, it is essential for the cell to have tight regulatory mechanisms for proper kinetochore assembly. Among the multiple proposed mechanisms, post-translational modification of outer kinetochore proteins emerged as an important regulatory mechanism to control kinetochore assembly and disassembly. For instance, CDK1 plays a central role in kinetochore function by phosphorylating kinetochore proteins such as CENP-T (20), which is important for recruitment of the Ndc80 complex to kinetochore during early mitotic phase. Aurora B is another important kinase that controls kinetochore–microtubule attachments by directly phosphorylating the KMN network and the Ska complex proteins at the kinetochore (21). Aurora B-mediated phosphorylation of Ndc80 in the KMN network reduces its microtubule-binding affinity, which prevents inaccurate attachment of kinetochores to microtubules. Aurora B also phosphorylates the Mis12 complex, which facilitates kinetochore assembly by forming a stable Mis12–CENP–C complex. In contrast, another mitotic kinase PLK1 was also shown to localize to kinetochores and to directly participate in stabilizing microtubule–kinetochore attachments by phosphorylating substrates such as INCENP and BubR1 (22). Although roles of various kinases in kinetochore function have been established, the importance of dephosphorylation events has not been well documented during this process.

In this study, we provided multiple lines of evidence supporting an essential role of a phosphatase PHLPP1 in kinetochore assembly by regulating SGT1 protein levels. Previous studies showed that SGT1 plays an essential role in kinetochore assembly, but mechanisms to control its functions at kinetochores are not well defined. Previously, CK2 (casein kinase 2) was shown to negatively regulate SGT1 by phosphorylating it at serine 361 (in yeast), thus inhibiting SGT1 dimerization and affecting the kinetochore assembly (23). In contrast, PLK1 was shown to phosphorylate SGT1 at serine 331 (corresponding to Ser-361 in yeast and Ser-299 in human SGT1A isoform) at kinetochores in humans, which is required for recruitment of outer kinetochore proteins Mis12 and NDC80 that help in the establishment of microtubule-binding sites (24). Thus, the role of phosphorylation in mediating SGT1 functions at the kinetochore is controversial. Interestingly, we did not find these residues to be phosphorylated by SGT1 in our studies, possibly due to low abundance of these phosphorylated peptides in cells, or they might be dynamically removed by active phosphatases. But nonetheless, we found four additional SGT1 phosphorylation sites that are required for its association with E3 ligase RNF41. PHLPP1 dephosphorylates these residues and prevents SGT1 association with RNF41, thereby helping SGT1 accumulation. We presume that this phosphorylation might have an important role in disassembly of kinetochores during the mitotic exit as we found reduced association of PHLPP1 with SGT1 during the late stages of mitosis. In fact, an earlier study has shown that SGT1 enhances degradation of PHLPP1 by promoting PHLPP1 association with its E3 ligase β-TrCP (25). Thus, we speculate that there might exist a negative feedback loop between PHLPP1–SGT1 during kinetochore assembly/disassembly cycles. PHLPP1 stabilizes SGT1 during kinetochore formation and then SGT1 might promote PHLPP1 degradation at later stages of mitosis where disassembly occurs. Although we identified PHLPP1 as a phosphatase for removal of the phosphate groups, it would be critical in future studies to identify the kinases that phosphorylate these residues and maintain optimal levels of SGT1 in cells.

In contrast, all the tumor suppressor activities of PHLPP1 have so far been attributed to its role in inhibiting various proliferation and survival signaling pathways. Our study provides an additional mechanism for PHLPP1 tumor suppressor function where loss of PHLPP1 resulted in multiple mitotic defects that subsequently lead to genomic instability. Further studies are required to uncover other possible substrates of PHLPP1 in mitosis, which may help in substantiating the role of PHLPP1 in controlling genomic stability.

## Experimental procedures

### Plasmids

Full-length PHLPP1, SGT1, RNF41, and PHLPP1 devoid of the phosphatase domain were cloned into SFB triple-tagged (S-protein/FLAG/streptavidin-binding protein) and Myc destination vectors using the Gateway cloning system (Invitrogen). PHLPP1 was also cloned into an HA destination vector. The point mutations for PHLPP, SGT1, and RNF41 were generated by PCR-based site-directed mutagenesis. SGT1 plasmid was a kind gift from Dr. Anna Filipek. Human RNF41 cDNA was procured from the DNASU repository.

### Antibodies

Anti-PHLPP1 (A300-660A from Bethyl Laboratories and 07-1341 from Millipore), anti-SGT1 (A302-944A from Bethyl Laboratories and Ab30931 from Abcam), anti-RNF41 (A300-048A from Bethyl Laboratories), anti-CENP-A (Ab13939 from Abcam), anti-CENP-E (Ab5093 from Abcam), anti-HEC1 (Ab3613 from Abcam) anti-Myc, clone 9E10 (SC-40 from Santa Cruz Biotechnology), anti-histone H3 (05928 from Millipore), anti-γ-tubulin (T5326 from Sigma), anti-α-tubulin (T6074 from Sigma), anti-FLAG (F3165 from Sigma), anti-actin (A5441 from Sigma), anti-HA (A190-108A from Bethyl Laboratories), and anti-ubiquitin (05-944 from Millipore) antibodies were used in this study.

### Tandem affinity purification

SGT1-associated proteins were isolated by using tandem affinity purification as described before (26). Briefly, 293T cells expressing SFB triple-tagged SGT1 were lysed with NETN buffer (20 mm Tris-HCl, pH 8.0, 100 mm NaCl, 1 mm EDTA, 0.5% Nonidet P-40) containing 50 mm β-glycerophosphate, 10 mm NaF, 1 μg/ml of each pepstatin A, and aprotinin on ice for 30 min. After removal of cell debris by centrifugation, the cell lysates were incubated with streptavidin-Sepharose beads (Amersham Biosciences) for 1 h at 4 °C. The bound proteins were washed three times with NETN and then eluted with 2 mg/ml biotin (Sigma) for 60 min at 4 °C. The eluates were incubated with S-protein-agarose beads (Novagen) for 1 h at 4 °C and then washed three times with NETN. The proteins bound to S-protein-agarose beads were eluted by boiling in SDS-loading dye and then resolved by SDS-PAGE. The identities of eluted proteins were revealed by mass spectrometry analysis performed by the Taplin Biological Mass Spectrometry Facility at Harvard University.

### Cell transfections, immunoprecipitation, and immunoblotting

HEK293T and HeLa cells were maintained in RPMI 1640 medium supplemented with 10% FBS and 1% penicillin and streptomycin, and the cells were transfected with various plasmids using polyethyleneimine transfection reagent. For immunoprecipitation assays, cells were lysed with NETN buffer. The whole-cell lysates obtained by centrifugation (with equal concentration of protein in different samples) were incubated with 2 μg of specified antibody bound to either protein A or protein G-Sepharose beads (Amersham Biosciences) for 1 h at 4 °C. The immunocomplexes were then washed with NETN buffer four times and applied to SDS-PAGE. Immunoblotting was performed according to the standard protocols.

### In vivo ubiquitination assay

HEK293T and HeLa cells were transfected with various combinations of plasmids, and 24 h after transfection, cells were treated with MG132 (10 μm) for 6 h. The whole-cell extracts prepared by NETN lysis under denaturing conditions were subjected to immunoprecipitation of the substrate protein. The levels of ubiquitinated protein were then detected by immunoblotting with ubiquitin antibody.

### RNA interference

The vector containing PHLPP1 shRNA-1 (5′-CCAGACTTACTACATTTGCTT-3′), PHLPP1 shRNA-2 (5′-CGAGGTCTTTCCCGAAGTTAT-3′), and RNF41 shRNA (5′-CGAAGATCTTATCTGCCCTAT-3′ was transfected, and 48 h post-transfection, the cells were collected. The efficiency of knockdown was checked by immunoblotting with specific antibodies.

### Cycloheximide chase assay

Cells were transfected with various combinations of plasmids, and 24 h post-transfection cycloheximide (50 μg/ml) was added. Cells were harvested at different time points, and protein levels were determined by immunoblotting after loading an equal concentration of protein per lane.

### In vitro phosphatase assay

GST SGT1 was bacterially purified, and an *in vitro* kinase reaction was performed by incubating SGT1 with 1 mm ATP, HEK293T cell lysate, and reaction buffer (25 mm HEPES, pH 7.5, 25 mm β-glycerophosphate, 25 mm MgCl_2_, 2 mm DTT, and 0.1 mm sodium orthovanadate) at 37 °C for 90 min. Following the incubation, beads were washed twice with NETN buffer. The phosphorylated SGT1 was incubated with bacterially purified MBP PHLPP1 WT and PHLPP1 D901N mutant in reaction buffer (25 mm HEPES, pH 7.4, 1 mm EDTA, and 10 mm DTT) at 30 °C for 60 min. The released phosphate was detected using malachite green assay kit (Cayman) by measuring the absorbance at 620 nm.

### pIMAGO-based detection of phosphorylation

GST-SGT1 was bacterially purified and phosphorylated *in vitro* by using 293T cell lysate. The phosphorylated SGT1 was incubated with bacterially purified phosphatases (MBP-tagged PHLPP1, D901N, mutant or PTEN) for 30 min. Later the samples were boiled, run on SDS-PAGE, and followed by transfer onto PVDF membrane. After incubating the membrane with blocking buffer (1 h), pIMAGO-Biotin reagent (Tymora Analytical) was added and further incubated for 1 h. After three washes (5 min each) with wash buffer, the membrane was incubated with avidin-HRP for 1 h. After washing three times with 1× TBST, phosphorylation signals were detected by ECL reagent. The phosphorylation status of SFB-tagged SGT1 wild type and SGT1 4A mutant in cells was also detected by immunoblotting with pIMAGO reagent.

### Immunofluorescence assay

HeLa cells were grown on coverslips and fixed with 3% paraformaldehyde solution in PBS at room temperature for 15 min. Permeabilization of the cells was carried out with 0.5% Triton X-100 buffer containing 20 mm HEPES, pH 7.4, 50 mm NaCl, 3 mm MgCl_2_, and 300 mm sucrose at room temperature for 5 min, and cells were incubated with 1% BSA for blocking at room temperature for 60 min. After this step, cells were washed with PBS and incubated with primary antibodies for 2 h at room temperature followed by washing twice with 1× PBS. Cells were then incubated with FITC or rhodamine-conjugated secondary antibodies at room temperature for 60 min followed by washing twice with 1× PBS. Nuclei were stained with 4′,6-diamidino-2-phenylindole (DAPI). After a final wash with PBS, coverslips were mounted with glycerin-containing *para*-phenylenediamine, and imaging was done using a confocal microscope (LSM Meta 510, Zeiss).

### Time-lapse video microscopy for live cells

HeLa cells were transfected with control and PHLPP1 shRNA, and 72 h after the transfection, cells were arrested using thymidine for 16 h. Later the cells were released from thymidine using fresh medium, and then image acquisition was performed with a Nikon A1R microscope equipped with a ×20 lens and enclosed in a chamber to maintain temperature for live cells. During imaging, cells were maintained in RPMI 1640 medium at 37 °C, and images were acquired at 1-min intervals with Nikon software. U2OS cells stably expressing H2B-mCherry were used in time-lapse imaging for mitotic stage-specific experiments.

### Statistical analysis

Data from at least three sets of independent experiments were plotted in the graphs by including standard deviation. The *p* values were determined by Student's *t* test using GraphPad software.

## Author contributions

S. M. and N. R. G. designed the experiments, analyzed the data, and wrote the manuscript. N. R. G. performed all the experiments.

## Supplementary Material

Supplemental Data

## Footnotes

3 The abbreviations used are:

SBP: streptavidin-binding peptide
MBP: maltose-binding protein
MT: microtubule
Ub: ubiquitin
S6K: S6 kinase 1
PTEN: phosphatase and tensin homolog.
