# Supplementary material for: Interplay between the phosphatase PHLPP1 and E3 ligase RNF41 stimulates proper kinetochore assembly via the outer-kinetochore protein SGT1
Source: J Biol Chem. 2017 Jul 10;292(34):13947–58. doi: 10.1074/jbc.M117.782896 (PMC5572923; doi:10.1074/jbc.M117.782896)
Supplement: Supplemental Data [file supp_292_34_13947__index.html]

Interplay between the phosphatase PHLPP1 and an E3 ligase RNF41 stimulates proper kinetochore assembly via the outer-kinetochore protein SGT1 — Interplay between the phosphatase PHLPP1 and E3 ligase RNF41 stimulates proper kinetochore assembly via the outer-kinetochore protein SGT1 — PHLPP1 and kinetochore assembly — Supplemental Data 

# Interplay between the phosphatase PHLPP1 and E3 ligase RNF41 stimulates proper kinetochore assembly via the outer-kinetochore protein SGT1

## Supplemental Data

- Supplementary data (.pdf, 826 KB) - Figure S1: Identification of phosphorylated sites on SGT1 Table S1: List of SGT1 associated proteins identified by mass spectrometric analysis
