# Supplementary material for: Interplay between the phosphatase PHLPP1 and E3 ligase RNF41 stimulates proper kinetochore assembly via the outer-kinetochore protein SGT1
Source: J Biol Chem. 2017 Jul 10;292(34):13947–58. doi: 10.1074/jbc.M117.782896 (PMC5572923; doi:10.1074/jbc.M117.782896)
Supplement: Supplemental Data [file 10.1074_M117.782896_jbc.M117.782896-1.pdf]

Interplay between the phosphatase PHLPP1 and an E3 ligase RNF41 stimulates proper kinetochore assembly via the outer-kinetochore protein SGT1

Narmadha Reddy Gangula[1] & Subbareddy Maddika[1*]

[1]Laboratory of Cell Death & Cell Survival, Centre for DNA Fingerprinting and Diagnostics (CDFD), Nampally, Hyderabad 500001, INDIA

Running title: PHLPP1 and kinetochore assembly

*To whom correspondence should be addressed

Dr. Subbareddy Maddika, Tel: +91-40-24749353, Fax: +91-40-24749448,

E-mail: msreddy@cdfd.org.in

This file contains supplementary figure S1 and table S1

Figure S1

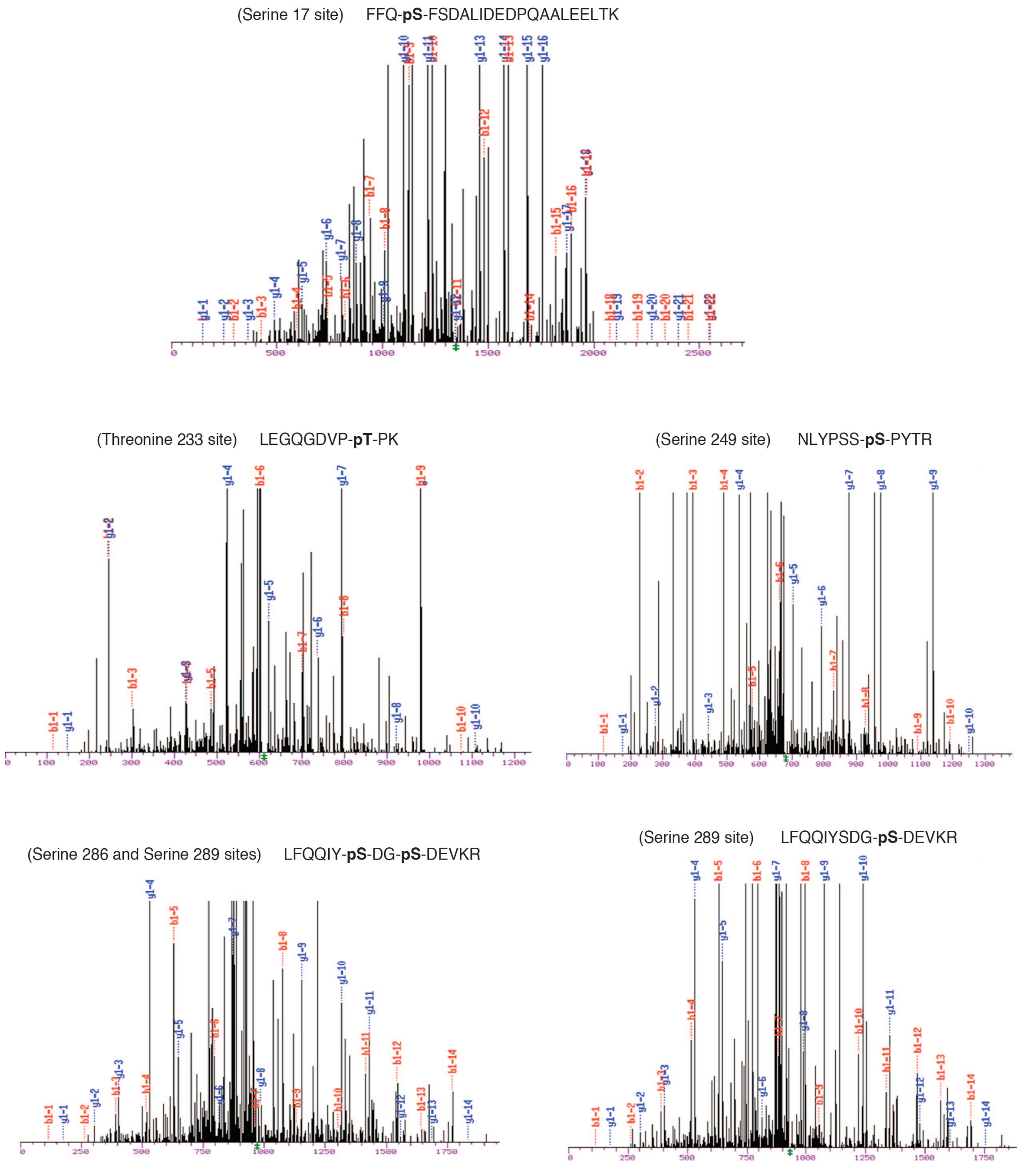

Figure S1: Identification of phosphorylated sites on SGT1. MS-MS spectra of phosphorylated SGT1 peptides were shown. The peptide sequences along with the phosphorylated residue (indicated in bold) identified in each of the peptide sequence were indicated.

Table S1: List of SGT1 associated proteins identified by mass spectrometric analysis

PROTEIN	TOTAL PEPTIDES
**Suppressor of G2 Allele Of SKP1 (SGT1)**	**1374**
Epiplakin 1 (EPPK1)	50
Erbb2 Interacting Protein (ERBB2IP)	47
Heat Shock Protein Family A (Hsp70) Member 8 (HSPA8)	59
Acetyl-CoA Carboxylase Alpha (ACACA)	27
Scribbled Planar Cell Polarity Protein (SCRIB)	27
Heat Shock Protein Family A (Hsp70) Member 1A (HSPA1A)	35
Peroxiredoxin 1 (PRDX1)	25
Chaperonin Containing TCP1 Subunit 2 (CCT2)	23
Heat Shock Protein Family A (Hsp70) Member 9 (HSPA9)	22
Centrosomal Protein 97 (CEP97)	22
Pyruvate Carboxylase (PC)	16
Tubulin Alpha 1a (TUBA1A)	24
Tubulin Beta 2A Class IIa (TUBB2A)	18
Heat Shock Protein Family A (Hsp70) Member 1 Like (HSPA1L)	21
Heat Shock Protein 90 Alpha Family Class A Member 1 (HSP90AA1)	18
Heat Shock Protein 90 Alpha Family Class B Member 1 (HSP90AB1)	18
Eukaryotic Translation Elongation Factor 1 Alpha 1 (EEF1A1)	14
Mitogen-Activated Protein Kinase 1 (MAPK1)	14
Heat Shock Protein 90 Alpha Family Class B Member 3, Pseudogene (HSP90AB3P)	14
Heat Shock Protein Family A (Hsp70) Member 5 (HSPA5)	13
Methylenetetrahydrofolate Dehydrogenase, Cyclohydrolase And Formyltetrahydrofolate Synthetase 1 (MTHFD1)	13
Leucine Rich Repeat Containing 40 (LRRC40)	10
T-Complex 1 (TCP1)	10

Pyruvate Kinase, Muscle (PKM)	10
Ribosomal Protein S3 (RPS3)	9
CTP Synthase 1 (CTPS1)	8
S-Phase Kinase-Associated Protein 1 (SKP1)	8
Stress Induced Phosphoprotein 1 (STIP1)	8
Carbamoyl-Phosphate Synthetase 2, Aspartate Transcarbamylase, And Dihydroorotase (CAD)	7
Solute Carrier Family 25 Member 11 (SLC25A11)	7
Chaperonin Containing TCP1 Subunit 3 (CCT3)	7
Heat Shock Protein Family A (Hsp70) Member 2 (HSPA2)	13
Tubulin Beta 4A Class IVa (TUBB4A)	9
Heat Shock Protein Family A (Hsp70) Member 4 (HSPA4)	7
**Ring Finger Protein 41 (RNF41)**	**7**
Cyclin Dependent Kinase 4 (CDK4)	7
Actin, Alpha 2, Smooth Muscle, Aorta (ACTA2)	7
Mitogen-Activated Protein Kinase 3 (MAPK3)	7
Solute Carrier Family 25 Member 4 (SLC25A4)	7
Leucine Rich Repeat Containing 1 (LRRC1)	6
Methylcrotonoyl-CoA Carboxylase 1 (MCCC1)	6
Solute Carrier Family 25 Member 13 (SLC25A13)	6
Tu Translation Elongation Factor, Mitochondrial (TUFM)	6
Solute Carrier Family 25 Member 3 (SLC25A3)	6
Protein Phosphatase 2 Regulatory Subunit Balpha (PPP2R2A)	6
Protein Kinase AMP-Activated Non-Catalytic Subunit Gamma 1 (PRKAG1)	6
Tubulin Beta 3 Class III (TUBB3)	8
Tubulin Beta Class I (TUBB)	7
Solute Carrier Family 25 Member 5 (SLC25A5)	7
Tubulin Beta 1 Class VI (TUBB1)	7
Calcyclin Binding Protein (CACYBP)	6
Heat Shock Protein Family D (Hsp60) Member 1 (HSPD1)	6

Inosine Monophosphate Dehydrogenase 2 (IMPDH2)	5
Lactotransferrin (LTF)	5
Eukaryotic Translation Initiation Factor 4A1 (EIF4A1)	5
RuvB Like AAA ATPase 1 (RUVBL1)	5
Eukaryotic Translation Elongation Factor 2 (EEF2)	5
Chaperonin Containing TCP1 Subunit 8 (CCT8)	5
Thioredoxin (TXN)	5
FK506 Binding Protein 5 (FKBP5)	5
Heat Shock Protein 90 Alpha Family Class B Member 2, Pseudogene (HSP90AB2P)	11
Complement C1q Binding Protein (C1QBP)	5
Actin Beta (ACTB)	5
Methylcrotonoyl-CoA Carboxylase 2 (MCCC2)	4
Tubulin Beta 6 Class V (TUBB6)	4
Nucleoporin 155 (NUP155)	4
Chaperonin Containing TCP1 Subunit 5 (CCT5)	4
Cofilin 1 (CFL1)	4
Glyceraldehyde-3-Phosphate Dehydrogenase (GAPDH)	4
Filamin A (FLNA)	4
Cullin Associated And Neddylation Dissociated 1 (CAND1)	4
Acyl-CoA Thioesterase 9 (ACOT9)	4
BPI Fold Containing Family A Member 1 (BPIFA1)	4
Eukaryotic Translation Elongation Factor 1 Gamma (EEF1G)	4
Plastin 3 (PLS3)	4
Phosphoglycerate Dehydrogenase (PHGDH)	4
Histone Cluster 2 H4 Family Member B (HIST1H4A)	4
ATPase Na+/K+ Transporting Subunit Alpha 1 (ATP1A1)	4
CCR4-NOT Transcription Complex Subunit 6 Like (CNOT6L)	4
Peroxiredoxin 2 (PRDX2)	4
BUB3, Mitotic Checkpoint Protein (BUB3)	4
Poly(RC) Binding Protein 1 (PCBP1)	4

Protein Phosphatase 5 Catalytic Subunit (PPP5C)	3
Translocase Of Inner Mitochondrial Membrane 50 (TIMM50)	3
Enolase 1 (ENO1)	3
Aminoadipate-Semialdehyde Dehydrogenase-Phosphopantetheinyl Transferase (AASDHPPT)	3
S-Phase Kinase-Associated Protein 2, E3 Ubiquitin Protein Ligase (SKP2)	3
Glycyl-TRNA Synthetase (GARS)	3
Annexin A2 (ANXA2)	3
Minichromosome Maintenance Complex Component 3 (MCM3)	3
NOP2/Sun RNA Methyltransferase Family Member 2 (NSUN2)	3
Propionyl-CoA Carboxylase Beta Subunit (PCCB)	3
Seryl-TRNA Synthetase 2, Mitochondrial (SARS2)	3
Cyclin Dependent Kinase Inhibitor 2A (CDKN2A)	3
Alpha-2-Glycoprotein 1, Zinc-Binding (AZGP1)	3
Neural Precursor Cell Expressed, Developmentally Down-Regulated 1 (NEDD1)	3
Ubiquitin Like Modifier Activating Enzyme 1 (UBA1)	3
